# The Case of Dr. Bartlett against the Erie County Medical Society

**Published:** 1864-05

**Authors:** 


### EDITORIAL DEPARTMENT.

### THE CASE OF DR. BARTLETT AGAINST THE ERIE COUNTY MEDICAL SOCIETY.

This case has occupied the attention of the Erie County Medical Society for several years, and finally came before the courts for adjudication. A connected account of it, therefore, may prove interesting to most members of the profession.

At the semi-annual meeting of the Erie County Medical Society held June 18th, 1859, an application for membership was received from Dr. Bartlett. This application, upon motion of Dr. Winne, was referred to a special committee, consisting of Drs. Winne, Rochester, Congar, Wyckoff and Burwell. At the next meeting, January 10th, 1860, the committee asked for further time to consider the application, which was granted. At the same meeting, however, later, the vote granting further time was reconsidered upon motion of Dr. Wilcox, and at the request of Dr. Rochester, one of the committee, the old committee was discharged and a new one appointed. The new committee consisted of five members, viz: Drs. Rochester, Congar, Gay, E. Storck and Burwell. At this meeting also a communication from Dr. Bartlett was read, renewing his application for membership and defining his position as a practitioner of medicine, especially denying the use of secret remedies. After conference the committee reported that they would meet Dr. Bartlett, for a hearing, one month from the day of the meeting of the society, and report at the next semi-annual meeting. Further time was again granted. Accordingly at the meeting in June following, the committee, through their chairman, Dr. Rochester, made the following report:

"That they have had an interview with the applicant, which established these facts: Dr. Bartlett graduated in the year 1854, from the Medical Department of the University of New York. During the same year he came to Buffalo and devoted his time to the treatment of diseases of the throat and lungs, advertising to that effect in the daily and weekly papers of the city. Moreover, he claimed in his advertisement to use the method of Robert Hunter of New York. The advertisement in its first form was discontinued upon his being notified of the impropriety of such a course, and one substituted which merely stated his name, residence, and specialty. He stated to the committee that he had never been guilty, consciously, of unprofessional conduct to any regular physician, and that he had never professed to employ secret or unusual remedies in the treatment of diseases. That he had once only held a consultation with an irregular practitioner, but had repeatedly declined to do so since. That it was his wish and intention to abandon special, and engage in general practice."

The report concluded with a recommendation that Dr. Bartlett be admitted to membership. It was signed by every member of the committee, except Dr. Burwell, who stated that he refused to sign it, and was opposed to granting the application. The report gave rise to an animated discussion; some members advocating and some opposing admission to membership, while others proposed a public recantation of errors as a condition of his being received as a member. The consideration of the matter was terminated by a vote of 15 yeas to 9 nays, to postpone indefinitely. The matter did not rest here, for at the afternoon session Dr. Rochester moved a re-consideration of the vote to postpone, which was carried, and the report came once more before the society. After some discussion a motion to adopt the report was lost by a vote of 6 nays to 3 yeas.

There appears to have been no further action in the case till the annual meeting, January 14th, 1862, when Dr. Cronyn *suggested*, as the record reads, the consideration of the application of Dr. Bartlett—the admission of members being in the regular order of business then under consideration. Dr. Whitney thought it would more properly come under the head of unfinished business. No motion appears to have been made, and nothing further done till the afternoon session, when the president *pro tem*, Dr. Samo, read a letter from Dr. Bartlett asking the society to take the application made by him two years before, under consideration. This letter is referred to in the opinion of the court given by Judge Daniels, which will be presently brought forward. It says the society took no notice of its requests. It might be inferred therefore, that there was not a disposition to deal fairly with him. But it should be borne in mind that the society had given him an interview through a committee, and there was no need to repeat it.

The letter will bear a few comments, being made so prominent by the above mention. It asks the society to lay aside all prejudice, consider his application in a kindly spirit, and not compel him to maintain an antagonism injurious and unpleasant to himself and it. He offers to explain any objections honestly entertained. There is an apparent candor about this which might to many carry the impression that he felt he had all the right on his side. It seems to imply on the part of the society, or at least some of its members, nothing else than prejudice, unkind spirit, bad temper, not strictly and solely a regard for the honor and welfare of the profession and society. In all such cases the individual should be carefully separated from the principle, and a man should not think because his wishes are denied, it is personal hostility—and so be tempted to call it prejudice or dishonesty. If the society had refused to notice this letter because it seemed to imply that its objections were personal and its motives dishonest, it would have been perfectly fair. But it was not so. There were no new facts and therefore no need of asking him or giving him an opportunity to explain. The objections were founded upon general, not personal grounds. We do not charge that Dr. Bartlett meant all that one might infer from his language, only that the society might without doing violence to the interpretation, take this view of it and consider it in no way proper in one who should ask it to overlook unprofessional conduct, not use language implying that its motives and spirit needed admonition.

A motion was made and carried to lay upon the table the communication, and we may suppose it still lies there. The application of Dr. Bartlett was not again brought before the society. The matter came before it in quite a different shape, the courts having been appealed to, to grant to the applicant the asserted rights, which he found the society not willing to recognize. In other words he took legal steps to compel that admission to membership, which he had failed to procure through the voluntary action of the society.

At a special meeting called March 6th, 1863, the president Dr. Winne, stated that a summons had been served upon the society to appear before Judge Davis at a special term of the Supreme Court, and show cause why Dr. Bartlett should not be admitted as a member of the society.—After some discussion, in which various propositions were made, viz: to employ an attorney and defend the suit; to take further time to consider the matter, and if there was good ground to believe Dr. Bartlett would practice regularly, admit him; to let the case go by default; to deny the jurisdiction of the court, and enter a protest, but give as little publicity to the matter as possible. Finally a motion prevailed to give the president power to employ an attorney and act as in his judgment seemed best. In accordance with the granted authority, the president employed counsel and defended the suit.

The case of Dr. Bartlett rested, it may be supposed, upon the general ground that bringing forward proofs of having graduated at a regular college, and having been regularly licensed to practice, he had a right to ask admission, slight admitted irregularities repented of and discontinued, not being a sufficient cause of rejection. The defence by the society was mainly alleged quackery, or, to soften the expression, unprofessional conduct, the advertisement mentioned above being the principal item of proof.

The result of the hearing was the issuing a peremptory mandamus for the admission of Dr. Bartlett to membership in the society. In order that the grounds of the decision may be more fully known it is copied entire:

"I am led by my reflections on this case to the conclusion that the application of the relator was improperly rejected. It is true that he had been guilty of acts of gross empiricism in the publication of the advertisement annexed to his affidavit, and had that publication been continued at the time of his application, I should regard the action of the society as entirely justifiable. But it appears without contradiction, that it had been abandoned for years, and the practice of the relator had been changed from the specialty mentioned in the advertisement to one of a general character.—There are no allegations made by defendant affecting the moral character of the relator, and I feel constrained to consider his course at the time of his first establishing himself in Buffalo, in the light of youthful indiscretions, rather than unpardonable offences. The *locus pœnitentiæ* was not shut against him, and his abandonment of his empirical conduct, for so long time before his application, affords satisfactory evidence of an intent to avail himself of the right and duty to reform. While I have no doubt that that the members of the society who voted for the exclusion were animated by a high sense of duty to their profession, yet they seem to me to have gone beyond their just powers in refusing him admission for acts which the relator had himself repudiated and abandoned. If the relator should resume his obnoxious course after his admission, he will be justly liable to the censure of the society, and ultimately to removal from its membership. But with the evidence of his present good conduct and of his adhesion to, and intention to conform to their rules and ethics, he was legally entitled to admission to the society, notwithstanding the professional errors of his early practice. The relator is entitled to the writ prayed for."

In order that the true character of the advertisement may be known it is here inserted, as first published, about May 1st, 1855:

"PROFESSIONAL NOTICE.—Dr. F. W. Bartlett, for the last three years associated with his brother-in-law, Dr. Robert Hunter, in the City of New York, in the treatmeat of diseases of the throat and lungs, has removed to Buffalo, and will continue to practice upon the method introduced by them, and successfully employed in the treatment of Bronchitis, Asthma and Consumption, in stages of progress heretofore deemed beyond the control of medicine. For the beneficial result of the treatment many residents of Buffalo bear grateful testimony. In consists mainly in the application of medicines in the condition of vapor, by means of an inhaling instrument, to the Bronchial and Pulmonary membranes, conjoined with such general treatment as experience has shown to be useful.

Dr. Bartlett refers to persons of the highest respectability in this, and the principal cities in the country, to substantiate his claims to the confidence of the public."

This advertisement was discontinued about January 12th, 1859, and the following inserted as a substitute:

"PROFESSIONAL NOTICE.—Dr. Frederic W. Bartlett may be consulted for all acute and chronic diseases of the throat and lungs at his office, 158 Pearl street."

This last was continued to near the time of Dr. B.'s application to the society for membership.

This was read at a special meeting, August 4th, 1863, and after some discussion it was voted that the president be requested to communicate with the president of the State Medical Society, and after consultation with him, that he be empowered to make appeal to the Term of the Supreme Court to be held in November, 1863, if he should judge it best to do so.

The president thought best to make an appeal, and the result of the second hearing before Justices Davis, Daniels and Grover was, the affirming of the previous order to admit.

At a special meeting, March 2d, 1864, the decision of the court, affirming the previous opinion of Judge Davis, was read. It was then voted that the president Dr. Wyckoff, Dr. Winne, and such other person as they may associate with themselves, be authorized to take any course they may think most conducive to the honor and welfare of the society. It should be stated that a motion had been previously made and withdrawn, to carry the case to the Court of Appeals. Acting under the discretionary power given them by the society, the gentlemen constituting the committee thought proper to carry the case to the Court of Appeals, where it now rests. In addition to the alleged irregular practice, the soceity claimed the right by its charter to exclude, as well as to admit. That it had the right to regulate its own affairs and to select its members, or rather to decide what specific acts should constitute a sufficient ground for denying the privilege of membership to one who, but for such disqualifications, would be considered as entitled to it. At the same time, while the society claimed to be acting within its legitimate powers in deciding what previous acts or circumstances should give it the right of exclusion, it would not assert that it should be exercised capriciously or against the admission of a regularly licensed physician against whom nothing could be alleged; whose conduct had been in every respect in accordance with the rules and ethics of the profession.—The society held that conduct which would have subjected a member to discipline or expulsion, would exclude a person applying for membership guilty of the same, from admission. The court held that such defence was not tenable in law. In order that the matter may be more plainly set forth the opinion of the court given by Judge Daniels, is here copied entire:

"The relator was regularly licensed as a physician and surgeon in this State in February, 1854, and has resided in the city of Buffalo, where he has practiced his profession since the early part of 1855, and is entitled to be admitted as a member of the Medical Society of Erie County, unless that right has been forfeited by the peculiar mode of practice adopted by him for the three or four years succeeding his settlement in Buffalo. That is insisted by the society to be the case, and to establish that result it relies upon the facts, that the relator devoted his professional attention to the treatment of diseases of the heart and lungs by medicated inhalations, and advertised his specialty in the public journals of the city.

The relator never has been a member of the Medical Society of Erie County, and there is nothing before the court to show that he has been a member of the State Medical Society. The statutory regulations of the practice of physic and surgery, and the by-laws and medical ethics both of the defendant and the State Medical Society have been referred to, for the purpose of sustaining the defendant in its objections. But while the statute provides that physicians and surgeons may be tried before the county court upon charges preferred against them for professional misconduct, gross ignorance, or immoral conduct, the proceedings are by its express language limited to those who are members of the medical society. They, and they alone, can be tried under its provisions. The relator therefore is not subject to these proceedings. The by-laws of the society are framed in the same manner. The offences prohibited and the punishments pronounced are alone for members, and no others, except in the single disability presently noticed.

For a member to conceal his art of curing diseases, to pretend to any superior skill or knowledge in the treatment of diseases, to consult or attend patients with a known quack, or any person not regularly authorized to practice, or with any physician who does not attach himself to the medical society, after residing in the county for one year, is punishable on the first conviction with a fine of five dollars, on the second ten dollars, on the third with expulsion. A member may also be expelled for neglecting or refusing to comply with the by-laws and regulations of the society, or of the State Medical Society. The object of the by-laws and of the code of ethics adopted by this society, or prescribed by the State Medical Society, is to regulate the conduct and define the duties of members and no others, except as others may be incidentally affected by the deportment of members towards them. For that purpose physicians neglecting to apply for membership after a year's residence in the county are excluded from consultations, and from attendance upon patients with its members. So also are those 'whose practice is based on an exclusive dogma to the rejection of the accumulated experience of the profession, and of the aids actually furnished by anatomy, physiology, and organic chemisty; or resort to public advertisements,' etc.

But the by-laws and ethics have not provided or declared that the physician who neglects to apply for membership within one year, or whose practice was based upon an exclusive dogma, or who had resorted to public advertisements, etc., shall be excluded from future membership. On the contrary the only disability imposed upon him is that which results from the duty of members of the society to avoid consultations and attendance upon patients with him. If the society have the power under the statute rendering it lawful for it to make by-laws and regulations relative to the admission of members, to exclude applicants upon those grounds, *they have not exercised it.*

But the relator for more than three years before his application to the Special Term for a mandamus, and for upwards of two years before the society finally refused his application for membership,' had discontinued both his public advertisements and his special practice, and had devoted his professional attention to general practice as an allopathic physician, with the intention of making that his future business. In that respect this case differs from that of *ex parte* Paine. The misconduct charged against the relator in that case was, that he was willing to practice on the allopathic or homœophathic system as patients desired, and was so engaged when the application to the society for membership and to the court for a mandamus were respectively made. The writ was denied because the court saw that the society could expel him for such professional misconduct as soon as he was admitted. The court declined to allow the writ where it could be rendered so completely ineffectual. The relator has, however, wholly discontinued that misconduct, which, if continued after membership, would violate the by-laws of the society, and might therefore result in his expulsion.

But it is claimed that the society acted judicially in denying membership to the relator. The objection is a good one, if it had any real foundation in fact. But so far from that being the case, the society had no jurisdiction over him. He had committed no offence against its by-laws, or the statute that gave it the power either to try or condemn him. If the society had been desirous of dealing with him as an offender against its laws, or the laws of the State, it should have given him the notice provided for in the statute, requiring him to become a member. Or he should have been received as a member upon his own application when he applied for that purpose, and then it could have enforced the by-laws against him, if he afterwards professionally misconducted himself. He offered in his last application for membership 'to explain any charges preferred, or to meet any objections honestly entertained.' But so far as the papers disclose no opportunity of that nature was extended by the society. Under the by-laws it could not be done, for they declare that 'no candidate shall be present until the question of his admission be determined by the Society.' The objections made to the admission of the relator to membership in the society are not well founded, and must be overruled. He has applied for the proper process to enforce his rights and shown himself entitled to it. The order of the Special Term should be affirmed."

In a future number some comments will be made upon the facts and principles involved in this case. L.

